# *Chlamydia pecorum* gastrointestinal tract infection associations with urogenital tract infections in the koala (*Phascolarctos cinereus*)

**DOI:** 10.1371/journal.pone.0206471

**Published:** 2018-11-01

**Authors:** Samuel Phillips, Amy Robbins, Joanne Loader, Jonathan Hanger, Rosemary Booth, Martina Jelocnik, Adam Polkinghorne, Peter Timms

**Affiliations:** 1 Genecology Research Centre, Faculty of Science, Health, Education and Engineering, The University of the Sunshine Coast, Queensland, Australia; 2 Endeavour Veterinary Ecology Pty Ltd, Toorbul, Australia; 3 Australia Zoo Wildlife Hospital, Beerwah, Australia; 4 Animal Research Centre, Faculty of Science, Health, Education and Engineering, The University of the Sunshine Coast, Queensland, Australia; University of California, San Francisco, Berkeley and the Childrens Hospital Oakland Research Institute, UNITED STATES

## Abstract

**Background:**

*Chlamydia* infects multiple sites within hosts, including the gastrointestinal tract (GIT). In certain hosts, gastrointestinal infection is linked to treatment avoidance and self-infection at disease susceptible sites. GIT *C*. *pecorum* has been detected in livestock and koalas, however GIT prevalence rates within the koala are yet to be established.

**Methods:**

Paired conjunctival, urogenital and rectal samples from 33 koalas were screened for *C*. *pecorum* and *C*. *pecorum* plasmid using 16S rRNA and CDS5-specific quantitative PCR assays, respectively. Amplicon sequencing of 359 bp *omp*A fragment was used to identify site-specific genotypes.

**Results:**

The overall *C*. *pecorum* prevalence collectively (healthy and clinically diseased koalas) was 51.5%, 57.6% and 42.4% in urogenital, conjunctival and gastrointestinal sites, respectively. Concurrent urogenital and rectal *Chlamydia* was identified in 14 koalas, with no cases of GIT only *Chlamydia* shedding. The *ompA* genotype G dominated the GIT positive samples, and genotypes A and E’ were dominant in urogenital tract (UGT) positive samples. Increases in *C*. *pecorum* plasmid per *C*. *pecorum* load (detected by PCR) showed clustering in the clinically diseased koala group (as assessed by scatter plot analysis). There was also a low correlation between plasmid positivity and *C*. *pecorum* infected animals at any site, with a prevalence of 47% UGT, 36% rectum and 40% faecal pellet.

**Conclusions:**

GIT *C*. *pecorum* PCR positivity suggests that koala GIT *C*. *pecorum* infections are common and occur regularly in animals with concurrent genital tract infections. GIT dominant genotypes were identified and do not appear to be related to plasmid positivity. Preliminary results indicated a possible association between *C*. *pecorum* plasmid load and clinical UGT disease.

## Background

In humans, *Chlamydia* infections have been directly linked to important diseases such as trachoma, pelvic inflammatory disease (PID) and tubular infertility [1–4]. Similar diseases are also reported in animals, caused by a range of veterinary chlamydial pathogens [5–10]. Beyond infections of the conjunctiva, urogenital and reproductive tracts, a growing body of literature is revealing that several *Chlamydia* species (*C*. *trachomatis*, *C*. *muridarum*, *C*. *suis* and *C*. *pecorum*) can survive within the host gastrointestinal tract (GIT), which can lead to gastrointestinal colonisation and a potential source of self-infection [11–18]. This infection potential is most well-established for the veterinary pathogen, *C*. *pecorum*, where rectal shedding rates can be greater than 30% in endemically infected sheep flocks and GIT infection is the initiator of systemic dissemination [19, 20]. Endemic rates of rectal colonisation and faecal shedding of *C*. *pecorum* have also been described in cattle, suggesting that GIT colonisation is an important source of transmission, and may lead to long-term adverse health effects [16–18].

In the koala (*Phascolarctos cinereus)*, *C*. *pecorum* is the leading cause of disease and a major contributing factor to individual population declines. *C*. *pecorum* transmission pathways in the koala have long been thought to be predominantly via sexual transmission [10, 21]. Pap-feeding, the process where the joey ingest maternal caecal material and the GIT is inoculated with microbes essential for browse digestion, has also been hypothesised as a vertical transmission route [22]. Despite the potential importance of *C*. *pecorum* GIT infection in the pathogenesis of *C*. *pecorum* disease in livestock, surprisingly little is known about this form of infection in koalas with few studies available [21, 23–26]. Two recent studies investigated the koala digestive microbiota by performing 16S rRNA pyrosequencing on hind-gut samples from healthy and diseased, wild and captive koalas, however, they reported no *Chlamydiaceae* sequences from any koala sampled [27, 28]. *Chlamydia* may not have been detected in these studies if examined samples lacked epithelial cells containing the intracellular chlamydial infection or as a result of the lowered sensitivity of the chosen (16S) sequencing target. Burach *et al*. (2014) found histological evidence of *Chlamydiaceae* in both the urogenital tracts (UGT) and GITs of nine koalas, being the first to suggest alternative modes of *Chlamydia* transmission [29]. More recently, a study in 2016 reported both matched and discordant *C*. *pecorum* genotypes between faecal pellets and UGT samples in five out of six Victorian koalas [25]. The longevity of GIT *Chlamydia* and the relationship between *C*. *pecorum* infections in the GIT, UGT and conjunctivae in koalas is otherwise unknown, challenging efforts to pathogenesis and the relevance of field sampling approaches that rely exclusively on the detection of *C*. *pecorum* shedding in koala faecal pellets [25].

The presence of C. *pecorum* plasmid carriage is another potential factor in pathogenesis, which has been identified in strains infecting koalas, sheep, cattle and pigs (Jelocnik et al., 2015) and originally described in koala strains in 1988 (Girjes et al.). In other species, plasmids expand the genetic diversity of the infecting *Chlamydia*, and can increase virulence and potential tissue tropism [30, 31]. Mouse models have demonstrated that the presence of a plasmid in *C*. *muridarum* species increases virulence, leading to ascending infection of the UGT and hydrosalpinx [32]. Shao and colleagues (2017), also demonstrated that only plasmid positive *C*. *muridarum* strains can colonise the mouse GIT [33]. However, the significance of the plasmid in the context of koala *C*. *pecorum* GIT infections is unknown and its role in koala *C*. *pecorum* pathogenesis is less clear, with two population studies finding contrasting rates of *C*. *pecorum* plasmid positivity in association with overt disease [34, 35].

Understanding the complexities of *C*. *pecorum* infections in the koala including the associated virulence factors, allows for accurate screening of koalas for chlamydial infections, helps in disease transmission modelling, and aids in the understanding of the effectiveness of future vaccines. In addition, identification of GIT reservoirs of *Chlamydia* in other species has implications for the screening protocols used to detect human chlamydial infections. The koala model can therefore be used to help understand chlamydial transmission and whether current treatment (antibiotics or vaccination) regimes are effective at clearing these GIT reservoirs.

We report here on an investigation into the prevalence of koala gastrointestinal *C*. *pecorum* shedding and the relationship to urogenital and ocular shedding, reporting on genotype disparities between anatomical sites and plasmid carriage involvement.

## Methods

### Ethics approval

Ethical approval for this study was granted by the Queensland Government, Department of Agriculture and Fisheries Animal Ethics Committee (AEC CA No. 2012/03/597 and 2013/09/719) and was performed under a Queensland Scientific Purposes Permit granted by Queensland Government, Department of Environment and Heritage Protection (SPP No. WISP11525212).

### Sample collection and DNA isolation

We analysed 163 samples from 29 (17 female, 12 male) apparently healthy koalas and four koalas (one female and three male) with signs of UGT disease, presented to two wildlife treatment facilities from four different regions of South East Queensland, Australia. Urogenital tract disease was diagnosed by a thorough veterinary examination which included a visual assessment for urinary incontinence (signs of “dirty tail/wet bottom”), sonographic evaluation of the bladder and reproductive tract, and cytological examination of the urine sediment. Urogenital (urethral for males and urogenital sinus for females), conjunctival, and rectal swabs and faecal pellets were collected from koalas and stored at -20°C, prior to transportation to the University of the Sunshine Coast (USC). Swabs were swirled in 500 μL of sterile PBS, and 200 mg of the faecal pellet was placed into 1 mL of Qiagen stool storage buffer (InhibitEX Buffer) and stored at -20°C until DNA extraction. For DNA extraction, 200 μL (PBS homogenate from swabs) or 600 μL (InhibitEX homogenate from faecal pellets) was processed using the Qiagen, QIAamp DNA Mini Kit (Venlo, The Netherlands) following the “DNA Purification from Blood or Body Fluids, Spin Protocol” or the “DNA Purification from Scat, Spin Protocol”. All DNA aliquots were stored at -20°C until further use.

### *C*. *pecorum* PCR detection and ompA genotyping

We performed real-time PCR (qPCR) for the detection of *C*. *pecorum* genomic DNA targeting a 204 bp fragment of the 16S rRNA gene [36]. Quantification was performed by plotting the crossing points against a standard curve produced using a serial dilution of known standards from 1 x 10^6^ to 1 x 10^2^ copies/μL [37]. To determine the genotype of the infecting strain, we amplified a 359 bp region of the *C*. *pecorum omp*A gene (between variable regions three and four) [36] and performed Sanger sequencing (Macrogen, South Korea) to determine the *C*. *pecorum* ompA genotype present in urogenital, rectal and faecal samples according to the scheme first outlined in Kollipara et al., (2013). No ompA typing was performed on conjunctival *C*. *pecorum* qPCR positive samples. Forward and reverse *omp*A sequences were trimmed for quality and combined into one contig using the Staden sequence analysis software. Resulting sequences were analysed by BLASTn to infer the *omp*A genotype. *C*. *pecorum* genotype results were then analysed for genotype prevalence and diversity between different anatomical sites from the same koala. PCR amplification of the koala β-actin gene was also performed on all samples as an internal control to identify any PCR inhibition and failed DNA extraction [38].

The prevalence of *C*. *pecorum* at each site was noted and then each site was directly compared for concurrent *C*. *pecorum* at multiple sites using 2x2 tables. Generation of correlation coefficients, confidence intervals and *P*-values for comparisons of quantified rectal (rectal swab or faecal pellet), UGT and conjunctival (either eye) results were performed using the statistical package R (version x64 3.2.4).

We performed qPCR detection for the *C*. *pecorum* plasmid targeting a 233 bp fragment of the of the *C*. *pecorum* plasmid (CDS5 or Pgp3 locus) on all UGT, rectal and faecal pellet samples. Using specific primers plasF– 5’–AATGGAAGGAGCTGTTGTC– 3’ and plasR– 5’–GATGTTGTTTCTGCATTAAGG– 3’ and Bio-Rad Sybr-green Itaq master mix, with an initial 95°C enzyme activation for 5 minutes, then 40 cycles of 95°C denaturation for 5 seconds, 57°C primer binding for 30 seconds and 72°C for 25 seconds with a fluorescence data capture. Finally, a melt profile was generated from 55°C to 95°C at 0.5°C per 2 seconds per step.

## Results

We analysed a total of 163 samples from 29 outwardly healthy koalas and from four koalas with clinical signs of UGT disease (rectal samples from two outwardly healthy koalas were not collected) (S1 Table). Using a *C*. *pecorum*-specific 16S rRNA qPCR assay, we detected *C*. *pecorum* shedding in the conjunctiva of 19 koalas (57.6%) (12 female and seven male), the UGT in 17 koalas (51.5%) (nine female and eight male), and the gastrointestinal site (rectal swab and/or faecal pellet) in 14 koalas (six female and eight male) (Table 1).

**Table 1:** Prevalence of *C*. *pecorum* at urogenital, ocular and rectal sites.

Site	Result	All Koalas (%)	Female Koalas (%)	Male Koalas (%)
Ocular (either eye)	Positive	19 (57.6)	12 (66.7)	7 (46.6)
	Negative	14 (42.4)	6 (33.3)	8 (53.3)
Urogenital	Positive	17 (51.5)	9 (50.0)	8 (53.3)
	Negative	16 (48.5)	9 (50.0)	7 (46.6)
Rectal (rectal swab or faecal pellet)	Positive	14 (42.4)	6 (33.3)	8 (53.3)
	Negative	19 (57.6)	12 (66.7)	7 (46.6)
Total number of koalas sampled		33	18	15

There was a moderate agreement (Cohen’s Kappa = 0.56 95%CI (25.0, 86.8)) of *C*. *pecorum* shedding between rectal swabs and faecal pellets (Table 2), with no isolated cases of GIT *C*. *pecorum* (from either site).

**Table 2:** Comparison of faecal pellet and rectal swab *C*. *pecorum* 16S rRNA PCR results.

	Rectal swab				
Faecal pellet	Negative	Positive	Total	Cohen's kappa	95%CI
Negative	18	4	22	0.55	0.25, 0.87
Positive	2	7	9		
Total	20	11	31		

We observed a very high correlation between infection at the GIT and UGT sites, with 14 of the 17 (82%) positive animals being positive at both sites with a similar chlamydial DNA load between sites (R = 0.86, *P* = <0.0001 and 95%CI (72.84, 92.76)) (Table 3).

**Table 3:** Concurrent *C*. *pecorum* detection between urogenital and rectal sites.

				**Urogenital**	
			**Male Rectal**	Negative	Positive
	**Urogenital**		Negative	7	0
**Rectal**	Negative	Positive	Positive	0	8
Negative	16	3			
Positive	0	14		**Urogenital**	
Correlation	r = 0.86 P = <0.0001		**Female Rectal**	Negative	Positive
	95%CI (72.84, 92.76)		Negative	9	3
			Positive	0	6

By comparison, only 33.3% of koalas had concurrent conjunctival and GIT *C*. *pecorum* infections (five males and six females). There was also a disparity in the chlamydial DNA load between GIT and conjunctival sites (R = 0.24, *P* = 0.19 and 95%CI (-11.75, 53.53)) (Table 4).

**Table 4:** Concurrent *C*. *pecorum* detection between conjunctival and rectal sites.

				**Ocular**	
			**Male Rectal**	Negative	Positive
	**Ocular**		Negative	5	2
**Rectal**	Negative	Positive	Positive	3	5
Negative	11	8			
Positive	3	11		**Ocular**	
Correlation	r = 0.24 P = 0.19		**Female Rectal**	Negative	Positive
	95%CI (-11.75, 53.53)		Negative	6	6
			Positive	0	6

Similarly, the level of co-infection at ocular and UGT sites was modest at 42.4% of koalas (five males and nine females), also with a disparity in the chlamydial DNA load between sites (R = 0.31, *P* = 0.08 and 95%CI (-3.79, 59.00)) (Table 5).

**Table 5:** Concurrent *C*. *pecorum* detection between ocular and urogenital sites.

				**Ocular**	
			**Male Urogenital**	Negative	Positive
	**Ocular**		Negative	5	2
**Urogenital**	Negative	Positive	Positive	3	5
Negative	11	5			
Positive	3	14		**Ocular**	
Correlation	r = 0.31 P = <0.08		**Female Urogenital**	Negative	Positive
	95%CI (-3.79, 59.00)		Negative	6	3
			Positive	0	9

**Fig 1:**
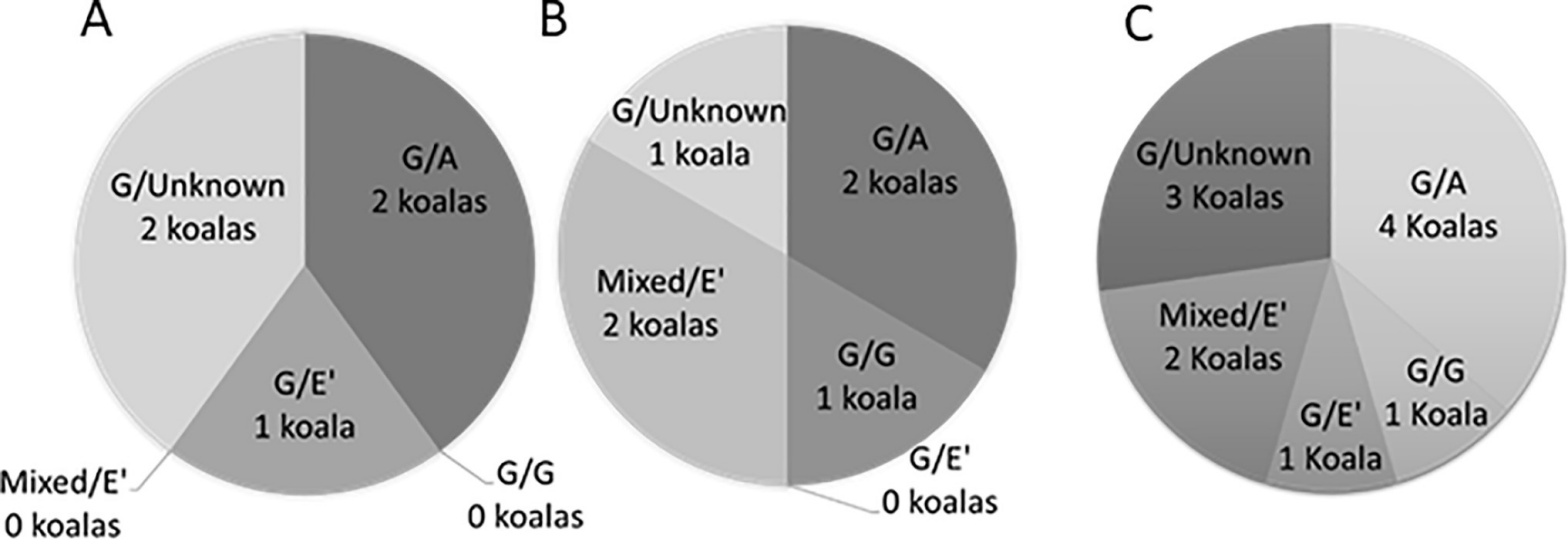
**ompA genotyping of *C*. *pecorum* 16SrRNA positive koalas indicating concurrent infections at the rectal and urogenital tracts, represented with rectal genotype first (rectal swab and faecal pellet combined) and urogenital genotype second,** A) Shows only mixed infections between the rectal and UGT sites occurring in female koalas, indicating only sexual transmission of *C*. *pecorum* B) Shows both mixed and identical genotypes infecting the rectal and urogenital sites of male koalas, indicating both sexual and faecal/genital transmission of *C*. *pecorum*. C) Overall prevalence of concurrent infections in all koalas.

**Fig 2:**
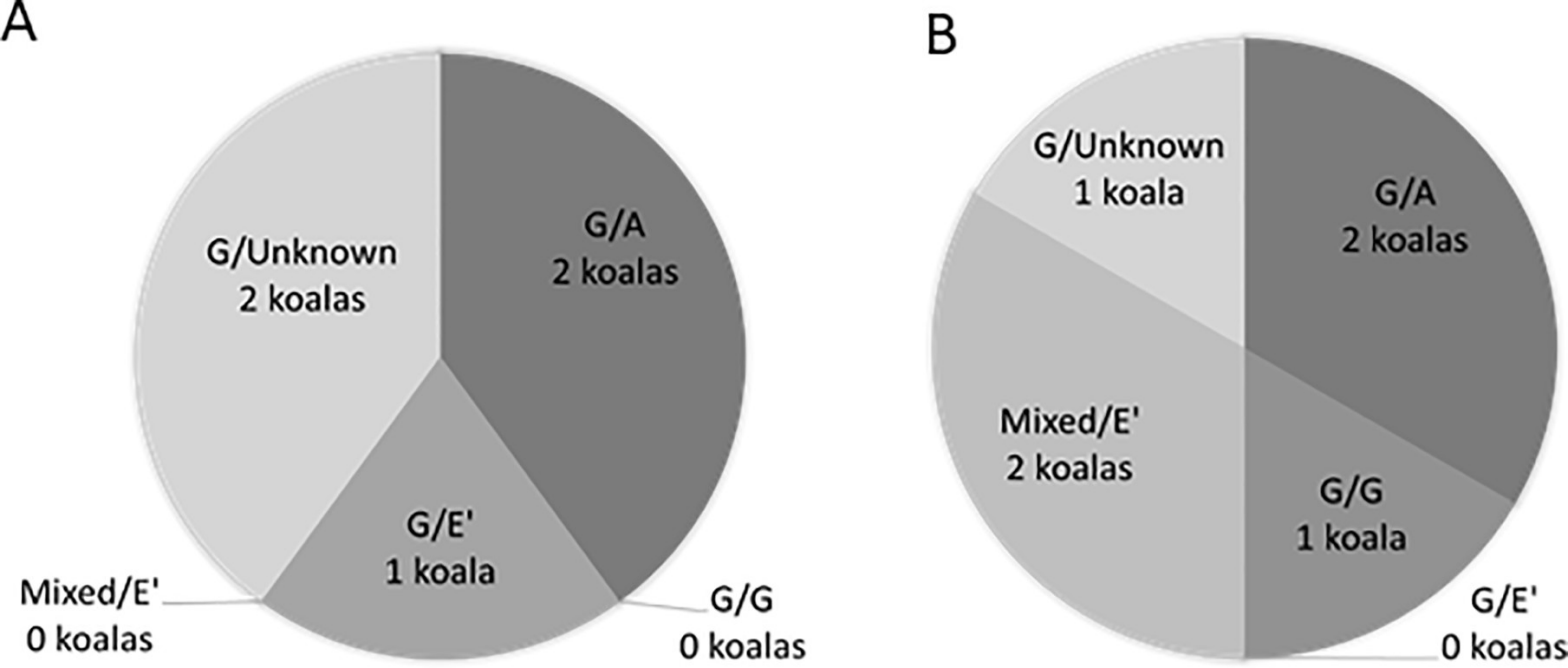
**ompA genotyping of *C*. *pecorum* 16SrRNA positive koalas indicating concurrent infections at the rectal and urogenital tracts, represented with rectal genotype first (rectal swab and faecal pellet combined) and urogenital genotype second**, A) Shows only mixed infections between the rectal and UGT sites occurring in female koalas, indicating only sexual transmission of *C*. *pecorum* B) Shows both mixed and identical genotypes infecting the rectal and urogenital sites of male koalas, indicating both sexual and faecal/genital transmission of *C*. *pecorum*.

To determine the infecting *C*. *pecorum* genotype at each individual site of infection in the same koala, we amplified and sequenced the variable domain 3–4 of the *C*. *pecorum omp*A gene (S1 Fig and S2 Table). Of the 12 positive rectal swabs, 11 were genotype G with one sample unable to be genotyped (Fig 1A). Of the 10 faecal pellets with detectable *C*. *pecorum*, seven were genotype G, one was genotype E’, one was genotype A and one sample was unable to be genotyped (Fig 1B). By comparison, of the 15 positive UGT samples, five were genotype A, three were genotype E’, one was genotype G and six samples were unable to be genotyped (Fig 1C). Comparison of *C*. *pecorum* genotypes of concurrent (GIT/urogenital) infections showed that female koalas only had mixed genotypes between sites, with GIT genotype G and UGT genotypes A and E’ identified (Fig 2A). Conversely, males had both mixed and identical genotypes between sites with GIT genotypes G, A and E’ and UGT genotypes G, A and E’ identified (Fig 2B).

**Fig 3:**
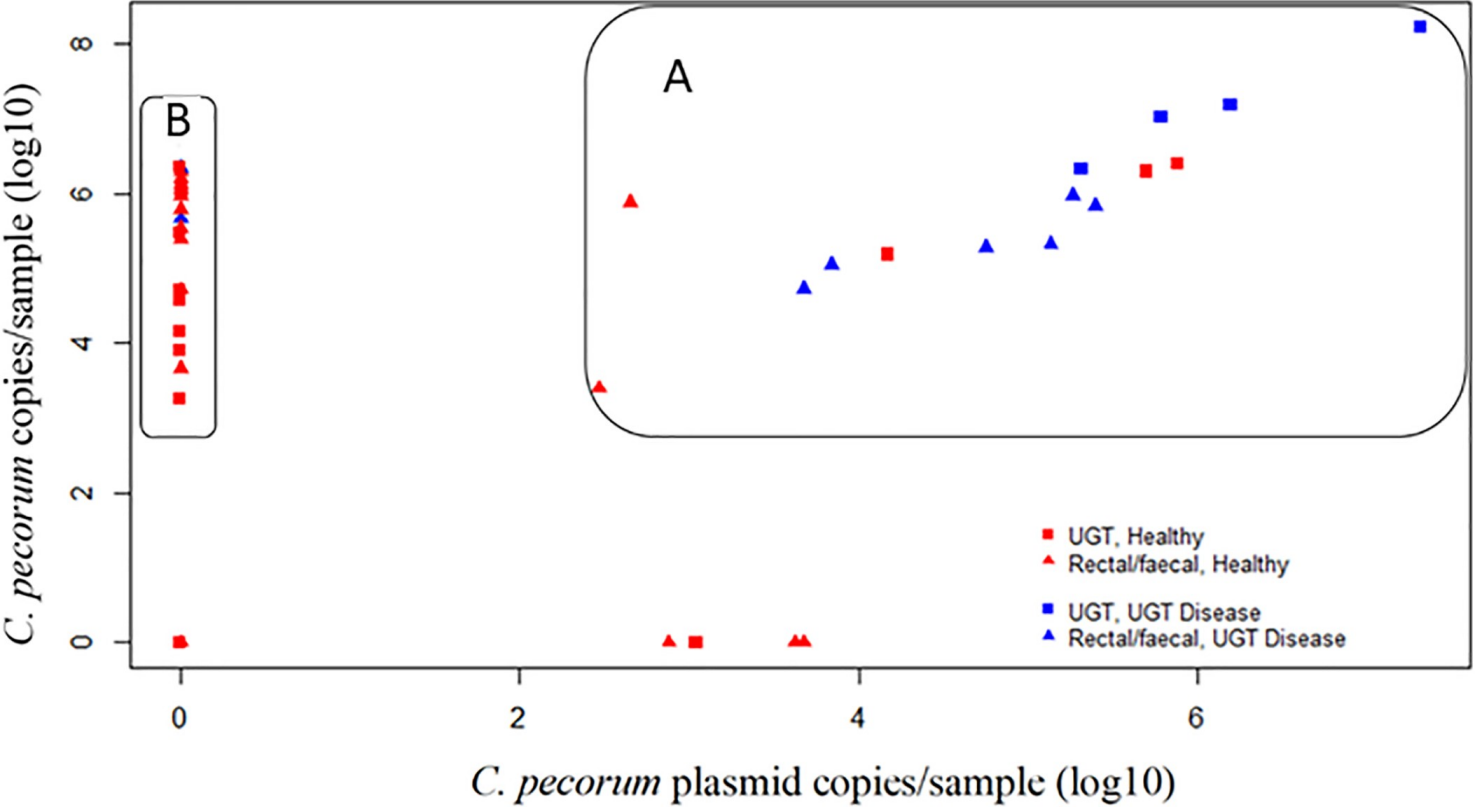
***C*. *pecorum* plasmid and 16SrRNA normalised (Log10) PCR load for the UGT, rectum and faecal pellets with current UGT disease indications,** A) Shows a second cluster of increasing plasmid PCR load dominated by koalas with signs of urogenital disease. B) Shows one cluster, indicating plasmid negative *C*. *pecorum* strains dominated by healthy koalas.

The presence of *C*. *pecorum* plasmid carriage was investigated by PCR to identify any associations between genotype site dominance and increased virulence. Analysis of *C*. *pecorum* positivity, plasmid positivity and koala UGT health status revealed two clusters of samples on a scatter plot (Fig 3). The first cluster (Fig 3A) was a cluster of high plasmid qPCR load (log10 > 2) with 66% (10/15 samples) of the samples from koalas with signs of UGT disease. The second cluster (Fig 3B) consisted of below detectable limits of plasmid (detected by qPCR) samples, consisting of 90% (19/21 samples) outwardly healthy koalas. There were no discernible differences between outwardly healthy koalas and koalas with signs of UGT disease when compared to *C*. *pecorum* load (detected by qPCR), with a DNA load range in each cluster of log10 between three and seven (with one outlier log10 > 8). There was also a low correlation between plasmid positivity and *C*. *pecorum* infected animals at any site, with a prevalence of 47% in the UGT, 36% in the rectum and 40% in faecal pellets (Table 6).

**Table 6:** Prevalence of *C*. *pecorum* plasmid positive strains.

	Overall plasmid positive (%)	16SrRNA and plasmid positive (%)
Urogenital	8 (24.2)	7 (46.6)
Rectal	5 (16.1)	4 (36.4)
Faecal pellet	6 (18.2)	4 (40.0)

Identification and quantification of the *C*. *pecorum* plasmid revealed that the identified rectal dominant *C*. *pecorum* genotype G is not associated with plasmid positivity, with only 18% of rectal positive samples being positive for the plasmid.

## Discussion

This study aimed to identify the prevalence of GIT *Chlamydia pecorum* infections in South East Queensland koalas and identify any relationships between infections at *Chlamydia* shedding sites through genotype analysis and plasmid positivity. The data presented indicates that *C*. *pecorum* is present within the GIT in 42% of the koalas sampled. Furthermore, GIT positivity was detected in 14/17 koalas with concurrent UGT *Chlamydia*. We found no association however, between conjunctival and GIT infections. Genotyping (*omp*A) results suggest genotype G is dominant within the GIT and that this dominance is not dependent on plasmid positivity. We also identified an association between plasmid positivity and UGT disease progression.

The high rate (82% of positive animals) of concurrent GIT and UGT chlamydial infections (as detected by qPCR) and relatively few UGT only infections (three female koalas) has been identified in other hosts and briefly koalas. Previous studies have reported that chlamydial species such as *C*. *muridarum* (*in vivo* lab studies), *C*. *gallinacea*, *C*. *suis* and recently *C*. *trachomatis*, can all colonise the GIT in mice, poultry, pigs and humans respectively [11–14]. Over the past 10 years four studies investigated *C*. *pecorum* colonisation at the GIT of both lambs and cattle [16, 17, 19, 20]. In addition, there have been four studies investigating koala GIT colonisation by *C*. *pecorum* which identified *Chlamydiaceae* DNA within mucosal tissues of the GIT and reported that *C*. *pecorum* can be detected from koala faecal pellets but not hind gut faecal material [25, 27–29].

Matching of genotypes between GIT and UGT sites showed that only two koalas had the same genotype at both sites (G/G and mixed/E’ (mixed = G/E’)) (Fig 2B). This suggests that perhaps genetically distinct strains have distinct tissue tropisms in the koala. While genotype G was the dominant genotype in the GIT, genotypes A and E’ were the dominant types at the UGT site. *C*. *pecorum* tissue tropisms have previously been identified in livestock, with *C*. *pecorum* multi locus sequence types 62, 63, 71, 78, 79, 80, 81 and 83 dominating the rectum and sequence types 23, 69, 72 and 82 dominating the conjunctiva in Australian sheep [19]. Tissue tropic strains of *Chlamydia* have previously been reported in humans with *C*. *trachomatis omp*A genotypes A to C dominating the ocular site and strains D to K dominating the genital site [4, 39].We used partial *omp*A gene to genotype our strains, and while this gene has been used extensively for *C*. *pecorum* genotyping in the past, [35, 36, 40, 41] it is unlikely that the major outer membrane protein is solely responsible for any tissue tropism. Recent reports, for example, indicate that *C*. *trachomatis* genotype G is rectal tropic due to three polymorphisms contained within the ORFs encoding for two Pmp proteins (CT144, CT154 and CT326)[42].

Previous studies have shown that not all strains of *C*. *pecorum* carry the plasmid and that the presence of the plasmid might correlate with virulence [34, 43]. We were able to identify 19 plasmid positive *C*. *pecorum* isolates in this study. Overall 20% of the samples contained a plasmid bearing *C*. *pecorum*, with a similar distribution between sampled sites (UGT 24%, rectal 16% and faecal pellet 18%). A strong association between UGT disease and plasmid positivity was identified, with 100% of koalas with UGT disease infected with plasmid positive chlamydial strains (Fig 3A). Furthermore, it was observed that a high plasmid load was associated with UGT disease. Studies in mouse models with *C*. *muridarum* have also identified this association, reporting that only plasmid positive *C*. *muridarum* strains were able to ascend the UGT and cause disease [32]. However, our results are based on only four koalas and further identification of plasmid positive strains from koalas with current UGT disease is needed to confirm these findings.

The correlation results in our study show that there is a significant probability that a koala with UGT *C*. *pecorum* will also have GIT *C*. *pecorum*, although with variation in infecting genotype. Further analysis of these results showed that 22% of males had an identical *Chlamydia* genotype at both the GIT and UGT sites, indicating that direct faecal genital contamination may be present in males, presumably associated with the anatomical positioning of the retracted penis. By comparison, in female koalas, there were no identified concurrent matching genotypes at the GIT and UGT sites. Furthermore, only females were identified with isolated UGT infections, further indicating the male anatomy is a source for faecal genital contamination.

Limitations to this study were the use of *C*. *pecorum* 16S rRNA targets, which have recently been identified as misidentifying non *C*. *pecorum* DNA targets [44]. However, by confirming all 16S rRNA samples using ompA as a secondary target we overcame the lowered specificity of this target.

## Conclusions

We found that genotypes dominant in rectal swabs and faecal pellets were often different from those recovered from UGT swabs in the same koala providing evidence for GIT infection, as opposed to contamination of rectal swabs by UGT shedding. This finding has clinical implications for the monitoring of healthy koalas for the presence of *Chlamydia* infections and also has implications for vaccine research, with the need to monitor vaccine effectiveness at all sites of infection, including the GIT, UGT and conjunctival sites.

Although our finding are preliminary, the presence of plasmid-bearing *C*. *pecorum* strains in the UGT correlates with urogenital disease, suggesting that this could be a critical risk factor in the development of UGT disease.

## Supporting information

S1 Table *C*. *pecorum* real time PCR quantified results and ompA (VD3/4) genotype at each anatomical site. (XLSX)

S1 Fig Maximum Parsimony analysis of taxa for all 29 ompA genotypes identified. (TIF)

S2 Table *C*. *pecorum* ompA (VD3/4) genotype sequences. (XLS)
