# Supplementary figures and images for: Chlamydia pecorum gastrointestinal tract infection associations with urogenital tract infections in the koala (Phascolarctos cinereus)
Source: PLoS One. 2018 Nov 1;13(11):e0206471. doi: 10.1371/journal.pone.0206471 (PMC6211709; doi:10.1371/journal.pone.0206471)

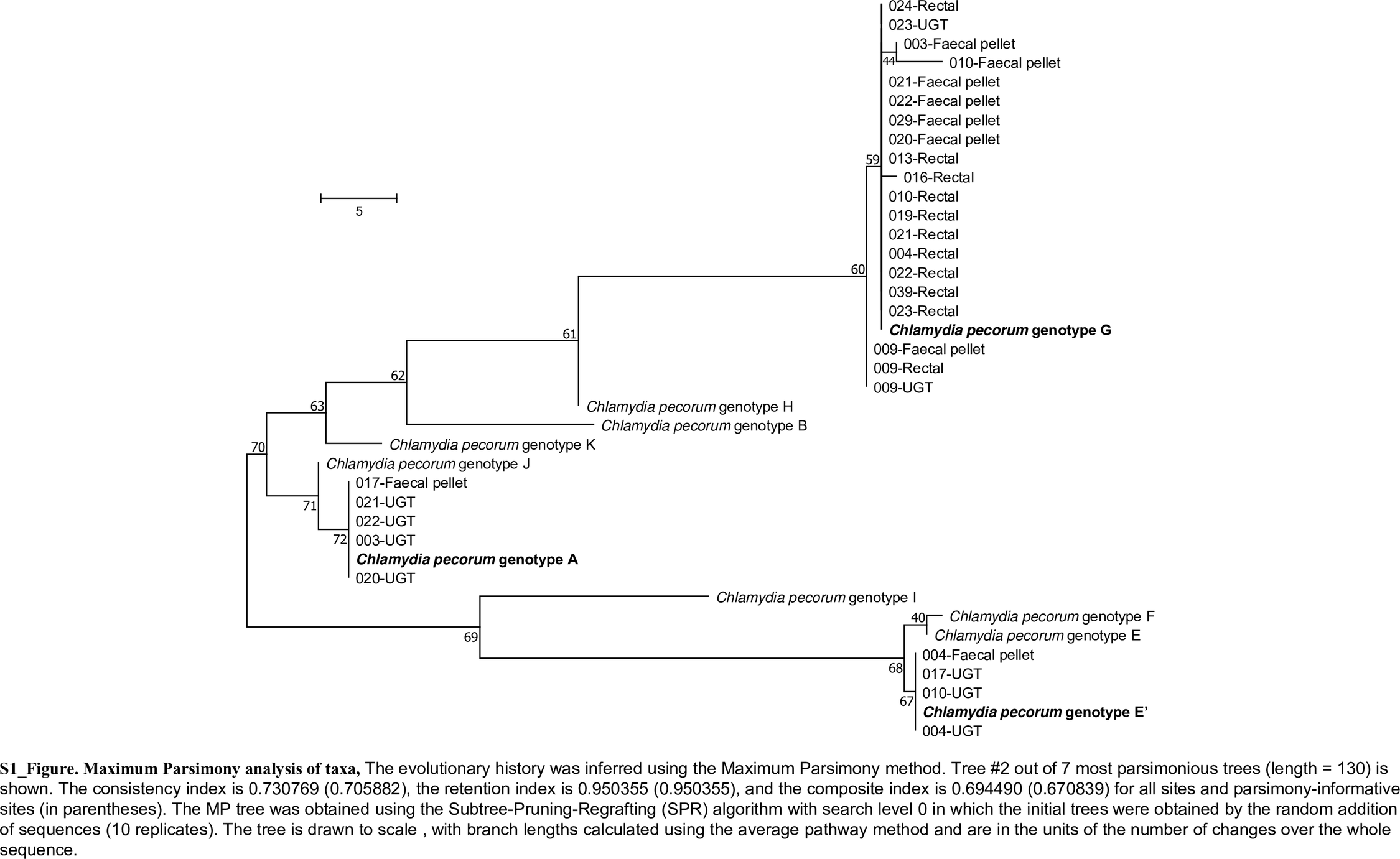

Supplement: S1 Fig — (TIF) [file pone.0206471.s002.tif]
